# Insulin Enhances the In Vitro Osteogenic Capacity of Flexor Tendon-Derived Progenitor Cells

**DOI:** 10.1155/2019/1602751

**Published:** 2019-12-27

**Authors:** Sushmitha S. Durgam, Nadine N. Altmann, Haley E. Coughlin, Audrey Rollins, Laura D. Hostnik

**Affiliations:** Department of Veterinary Clinical Sciences, College of Veterinary Medicine, The Ohio State University, 601 Vernon L. Tharp Street, Columbus, OH, 43210, USA

## Abstract

There is increased incidence of tendon disorders and decreased tendon healing capacity in people with diabetes mellitus (DM). Recent studies have also suggested pathological ossification in repair tendon of people with DM. Therefore, the objective of this study is to investigate the effects of insulin supplementation, an important pathophysiologic stimulus of DM, on tendon progenitor cell (TPC) proliferation and in vitro osteogenic capacity. Passage 3 TPCs were isolated from collagenase-digested, equine superficial digital flexor tendons. TPC proliferation was measured via MTT assay after 3 days of monolayer culture in medium supplemented with 0, 0.007, 0.07, and 0.7 nM insulin. In vitro osteogenic capacity of TPCs (Alizarin Red staining, osteogenic mRNA expression, and alkaline phosphatase bioactivity) was assessed with 0, 0.07, and 0.7 nM insulin-supplemented osteogenic induction medium. Insulin (0.7 nM) significantly increased TPC proliferation after 3 days of monolayer culture. Alizarin Red staining of insulin-treated TPC osteogenic cultures demonstrated robust cell aggregation and mineralized matrix secretion, in a dose-dependent manner. Runx2, alkaline phosphatase, and Osteonectin mRNA and alkaline phosphatase bioactivity of insulin-treated TPC cultures were significantly higher at day 14 of osteogenesis compared to untreated controls. Addition of picropodophyllin (PPP), a selective IGF-I receptor inhibitor, mitigated the increased osteogenic capacity of TPCs, indicating that IGF-I signaling plays an important role. Our findings indicate that hyperinsulinemia may alter TPC phenotype and subsequently impact the quality of repair tendon tissue.

## 1. Introduction

Tendinitis and tendinopathies are common and debilitating injuries in people [1–4]. Recent studies show that people with diabetes mellitus (DM) have increased incidence of tendon disorders and are mediated in part due to structural alterations [5, 6]. In addition, diabetic patients with tendon injuries have decreased tendon healing capacity and are at a greater risk for recurrent injury [5–7]. Increased tendon size [8, 9], disorganized collagen fibers [8, 9], and aberrant ossification in repair tendon [10, 11] are some pathologies reported in people with DM. Increased calcium deposition at Achilles tendon insertions has been observed in people with DM, which subsequently leads to tendon rupture. Pathological ossification within repair tendon reduces tendon elasticity, increases risk of repeat injury, and is a source of chronic pain and discomfort in people [12, 13]. While recent studies indicate increased tendon pathology in people with DM, the biological and cellular mechanisms responsible are poorly understood.

The cellular fraction of tendons, consisting of mature tenocytes and multipotent tendon progenitor cells (TPCs), is very small (<1%) but is responsible for the synthesis and turnover of extracellular matrix (ECM), which forms the bulk of tendon tissue [14]. Tendon progenitor cells demonstrate universal stem cell characteristics of clonogenicity, multipotency, and self-renewal, and their bioactivity is heavily dependent on their local environment/matrix interactions [14–16]. The in vitro and in vivo phenotypes of tendon progenitor cells in healthy and pathological tendon tissue have shown to be markedly different [15–17]. In vivo, TPCs in repair tendon are implicated in chondrodegeneration noted during healing [17, 18]. Similarly, the in vitro multipotency of TPCs isolated from injured tendon is restricted and shifts towards a chondroosteogenic phenotype [17, 19]. A recent study by Shi et al. demonstrated that TPCs isolated from DM have a decreased tenogenic phenotype and express higher levels of chondroosteogenic genes [20]. Taken together, aberrant differentiation of TPCs under the influence of pathological stimuli present in DM may be responsible for pathological calcified tissue present in injured/healing tendon.

Exogenous insulin is used in clinical management of DM. In addition, systemic hyperinsulinemia secondary to impaired insulin sensitivity can occur during early DM. While the tenogenic phenotype of TPCs in injured DM tendon tissue and during in vitro culture with high-glucose concentration is significantly decreased, the effects of high-insulin concentrations on TPCs have not been investigated [20, 21]. Both exogenous insulin and recombinant insulin-like growth factor-I (IGF-I) increase the in vitro osteogenic capacity of osteoblasts and periodontal ligament fibroblasts [22, 23]. Therefore, we hypothesize that exogenous insulin affects TPC proliferation and enhances their in vitro osteogenic capacity. In the first set of experiments, the effects of exogenous insulin (Humulin™ 0, 0.07, and 0.7 nM) on proliferation and in vitro osteogenic capacity of equine flexor tendon-derived TPCs were assessed. Secondly, in order to determine if differential effects of insulin on insulin/IGF-I signaling exist, osteogenesis experiments were conducted in the presence of insulin and picropodophyllin (PPP), a selective inhibitor of IGF-I receptor.

## 2. Methods

### 2.1. Tendon Progenitor Cell (TPC) Isolation

TPCs were isolated from superficial digital flexor tendons of three healthy (4-6 years of age) adult horses using protocols previously described [14–16, 24]. These cells were characterized via cell surface marker expression (CD 44^+^, CD29^+^, CD90^+^, and CD45^−^) and in vitro trilineage differentiation as previously described [15, 16, 24]. An intact forelimb superficial flexor tendon was harvested from young adult horses euthanized for reasons unrelated to musculoskeletal disease. A 1-2 cm length of the midsubstance tendon specimen, free of peritendinous tissue, was diced into 0.25 cm^3^ pieces and digested in 0.2% collagenase (Worthington) in DMEM supplemented with 2% fetal bovine serum (Gemini Biomedical) at 37°C for 16 hours. The released cells were isolated by filtration and centrifugation, and the cells were seeded at 500 cells/cm^2^ in monolayer cultures in high-glucose DMEM supplemented with 10% fetal bovine serum, 37.5 *μ*g/mL of ascorbic acid, 300 *μ*g of l-glutamine/mL 100 U of sodium penicillin/mL, and 100 *μ*g of streptomycin sulfate/mL (basal medium). These cells were seeded onto cell culture flasks and incubated at 37°C with 5% CO_2_ to enable colony formation. Medium was replaced every 3 days. Once discernible colonies were formed (>200 cells/colony), the cells were detached with 0.02% EDTA and 0.05% trypsin. Cell numbers were calculated by counting an aliquot of the resulting suspension using a haemocytometer and an inverted light microscope. Trypan blue dye exclusion was used to assess cell viability. The primary cell isolates were reseeded at 5 × 10^3^cells/cm^2^ and passaged twice at 80-90% confluence to expand cell numbers and enrich for progenitor cells.

### 2.2. Cell Proliferation

Passage 3 TPCs were plated at 3 × 10^3^ cells/cm^2^ in basal medium in 96-well plates. After 1-day culture, insulin (at 0 nM, 0.007 nM, 0.07 nM, and 0.7 nM concentrations; Humulin™ U-100, Lilly, USA) treatments were added and incubated for 3 days. Three replicate wells for each treatment group were used to measure the cell numbers via a mitochondrial metabolic assay (Cell Titer MTT 96 aqueous one solution cell proliferation assay, Promega) which was used in accordance with the manufacturer's instructions. In brief, 20 *μ*L of the assay reagent containing tetrazolium was added into each well of the 96-well plate containing 100 *μ*L of fresh media and incubated at 37°C for 2.5 hours. Absorbance was measured at 490 nm in a microplate reader (Tecan™ Infinite 200 PRO plate reader) to detect concentrations of the metabolic product, formazan. The mean value was calculated from replicate wells to provide a single data point. These optical density values from plated TPCs from each horse were reported.

### 2.3. Osteogenic Differentiation

Passage 3 TPCs were plated at 5 × 10^3^ cells/cm^2^ in 6-well cell culture plates and maintained in complete DMEM until they reached 80-90%% confluence. Complete DMEM was then substituted with osteogenic medium (complete DMEM supplemented with 10 mM *β*-glyceraldehyde-3-phosphate, 50 *μ*g/mL ascorbic acid, 100 nM dexamethasone) or osteogenic medium with 0.07 or 0.7 nM insulin (Humulin® U-100, Lilly, USA). The medium was replaced every 48-72 hours [15, 16]. The cultures were maintained for 14 days. In addition, TPC osteogenic cultures (with 0, 0.07, and 0.7 nM insulin) were also maintained with or without 100 nM picropodophyllin (PPP; Selleckchem, # S766802), a small molecule inhibitor of IGF-I receptor. Control osteogenic cultures were cultured (+/- insulin) with an equal amount of DMSO.

### 2.4. Alizarin Red Staining of Osteogenic Cultures

At days 0, 7, and 14 of osteogenic culture, the medium was aspirated from the cell cultures and the cell monolayers were fixed with 1 mL of 4% formalin at room temperature for 30 minutes. After fixation, the cell layers were washed three times with PBS. One mL of 2% Alizarin Red (Sigma-Aldrich) solution (2 g of Alizarin Red dye dissolved in 100 mL of Milli-Q water; pH adjusted to 4.2) was added to each well and incubated at room temperature for 15 minutes. The unbound stain was then removed, and the cells were washed 3-4 times with water until the rinse solution was clear. Mineral deposits within the cell layers stained bright red. Low-magnification (10x) images were obtained prior to osteogenic differentiation and at days 7 and 14 of osteogenic culture. An inverted light microscope (Leica Microsystems, Leica Application Suite-LAS-version 6.0) was used to assess mineralized matrix deposition.

### 2.5. RNA Isolation and Quantitative RT-PCR

Total RNA was isolated using a previously described protocol [15, 25]. The samples were homogenized in guanidinium thiocyanate-phenol-chloroform solution reagent (TRIzol, Invitrogen) according to the manufacturer's suggested protocol. The resultant pellet was purified using RNeasy silica columns that included on-column DNase digestion. The concentration of RNA was determined by measuring the absorbance at 260 nM (A260) and 320 nM (A320) in NanoDrop One/One® (Thermo Fisher Scientific). One *μ*g of RNA from each sample was reverse-transcribed (Superscript IV, Invitrogen) using oligo (dT) primers. Equine gene-specific primers were designed from published sequences in Genbank and using ClustalW multiple sequence alignment (available at http://www.ebi.ac.uk) (Table 1). Primer specificity was confirmed by cloning and sequencing the amplicons during optimization experiments, as previously described. PCR amplifications were catalyzed by Taq DNA polymerase (ABI QuantStudio 3™, Thermo Fisher Scientific) in the presence of SYBR green. Relative gene expression was quantified using the 2^-*ΔΔ*CT^ method, normalized to expression of the reference gene, elongation factor-1*α* (EF1*α*) [26].

### 2.6. Alkaline Phosphatase (ALP) Bioactivity Measurements

ALP bioactivity was measured as previously described [27]. Briefly, prior to and at day 14 of osteogenic culture, the cells were harvested in 300 *μ*L of lysis buffer (20 mM Tris HCl, 150 mM NaCl, and 1% Triton X-100). Each sample was homogenized, centrifuged at 600 rcf for 15 minutes at 4°C, and kept on ice for 30 minutes. The supernatants were assayed for ALP activity using a commercially available ALP assay kit (Wako Chemicals) that measured the conversion of p-nitrophenylphosphate to p-nitrophenol. The intensity of yellow generated by this reaction after 10 minutes of incubation was measured at 405 nm wavelength (Tecan® Infinite 200 PRO plate reader).

The relative ALP bioactivity of each sample was determined by normalizing the measured p-nitrophenol release/minute to the corresponding DNA content.

### 2.7. DNA Measurement

DNA content was measured using the Quant-iT PicoGreen dsDNA kit (Invitrogen). Samples were diluted 1 : 5 in 1X TE buffer (10 mM Tris HCl, 1 mM EDTA, pH 7.5). Serial dilutions of lambda DNA were used to generate a standard curve. Duplicate 100 *μ*L aliquots of each sample and the standards were transferred to a black 96-well microplate. One hundred *μ*L of PicoGreen reagent (1 *μ*L PicoGreen reagent diluted in 200 *μ*L of 1X TE buffer) was added to each sample and standard. The microplate was placed in the dark to prevent reagent photodegradation. Following 5 minutes of incubation, the fluorescence was measured at 485 nm wavelength (Tecan® Infinite 200 PRO plate reader).

### 2.8. Statistical Analysis

The normal distribution of quantitative data (relative mRNA expression, alkaline phosphatase activity) was confirmed using the Kolmogorov-Smirnov test using SigmaPlot 14 software (Systat Software®). The data were representative of at least three independent experiments, each done in triplicate. All results are expressed as the mean ± standard deviation. One-way ANOVA was used to compare 0.07 and 0.7 nM insulin supplementation with untreated osteogenic cultures at day 14 of osteogenesis. A *p* value of ≤0.05 was considered statistically significant.

## 3. Results

### 3.1. Insulin Increases In Vitro TPC Proliferation (Figure 1)

After 3 days of monolayer culture with insulin, TPC proliferation with 0.7 nM insulin was significantly increased (~2-fold, *p* = 0.003) compared to untreated control. There was no significant difference in TPC proliferation with 0.007 and 0.07 nM insulin compared to untreated control.

### 3.2. Insulin Increases Osteogenic Differentiation of TPCs In Vitro (Figure 2)

#### 3.2.1. Alizarin Red Staining

**Figure 2:**
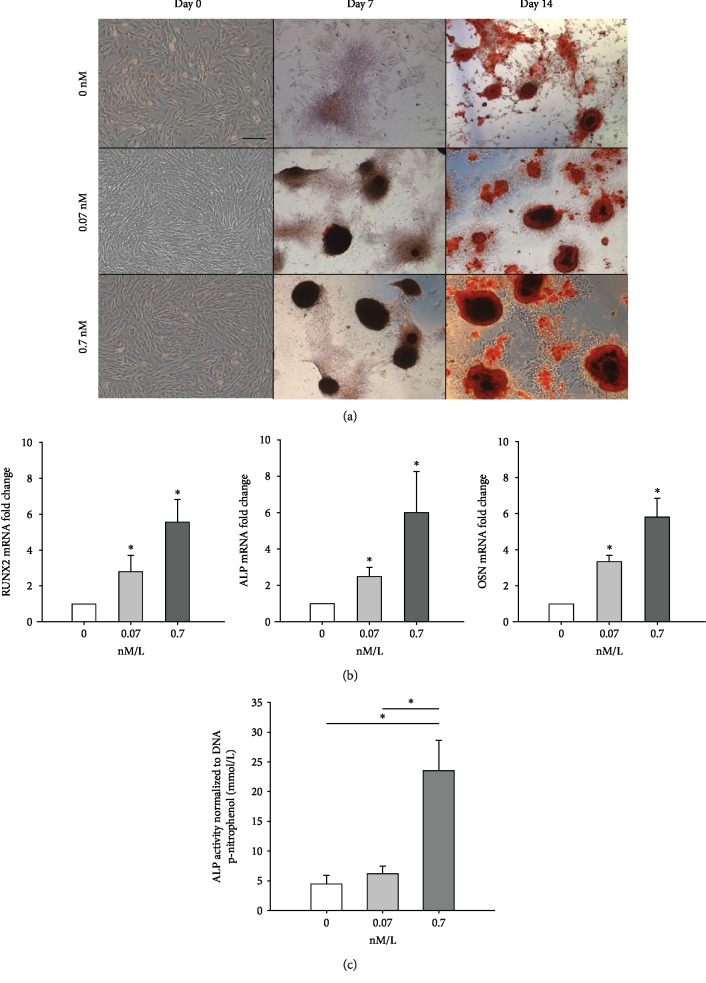
(a) Alizarin Red-stained TPC osteogenic cultures supplemented with 0, 0.07, and 0.7 nM/L insulin. Insulin promoted cell aggregation and mineralized matrix secretion as evidenced by intensity of Alizarin Red stain uptake. Scale bar = 500 microns. (b) mRNA levels (normalized to EF1a) of RUNX2, ALP, and Osteonectin (OSN) in TPC osteogenic cultures supplemented with 0, 0.07, and 0.7 nM/L insulin. Insulin treatment significantly increased osteogenic gene expression. ∗ represents a significant difference (*p* ≤ 0.05) between treatment groups. (c) Alkaline phosphatase bioactivity (normalized to total DNA content) of TPC osteogenic cultures supplemented with 0, 0.07, and 0.7 nM/L insulin. ∗ represents a significant increase in alkaline phosphatase activity (*p* ≤ 0.05).

Insulin increased aggregate formation and associated Alizarin Red staining in TPC osteogenic cultures (Figure 2(a)). Larger mineralized nodules were noted in osteogenic cultures containing 0.07 and 0.7 nM insulin, in a dose-dependent manner, at both day 7 and day 14 compared to untreated controls. By day 14, intense staining of mineralized nodules, indicative of robust osteogenic differentiation, was evident in osteogenic cultures containing insulin compared to untreated controls.

#### 3.2.2. Osteogenic Gene Expression

In day 14 of osteogenic cultures, the Runx2 mRNA level was significantly increased (*p* < 0.001) with increasing insulin concentrations (Figure 2(b)). The Runx2 mRNA level of osteogenic cultures with 0.7 nM insulin (5.5‐fold ± 1.25) was significantly higher than that with 0.07 nM insulin (2.8‐fold ± 0.9; *p* = 0.019) and untreated control (1; *p* = 0.002). There was no significant difference in the Runx2 mRNA level of 0.07 nM insulin and control osteogenic cultures. Similarly, the ALP mRNA level of day 14 of osteogenic cultures with 0.7 nM insulin (6.0‐fold ± 2.2) were significantly higher than that with 0.07 nM insulin (2.56‐fold ± 0.49; *p* = 0.045) and untreated control (1; *p* = 0.010). There was no significant difference in the ALP mRNA level of 0.07 nM insulin and control osteogenic cultures. The Osteonectin mRNA level in day 14 of osteogenic cultures with 0.7 nM insulin (5.8‐fold ± 1.04) was significantly higher than that with 0.07 nM insulin (3.3‐fold ± 0.35; *p* = 0.004) and untreated control (1; *p* < 0.001). The Osteonectin mRNA level in day 14 of osteogenic cultures with 0.07 nM insulin was significantly higher (*p* = 0.003) than untreated control.

#### 3.2.3. Alkaline Phosphatase (ALP) Activity

Alkaline phosphatase bioactivities in the osteogenic cultures were consistent with the transcriptional outcomes reported above. ALP bioactivity (mmol/L p-nitrophenol) of day 14 of osteogenic culture with 0.7 nM insulin (23.0 + 4.24) was significantly higher than that with 0.07 nM insulin (6.25 ± 1.49; *p* = 0.009) and untreated control (4.47 + 1.73; *p* = 0.009). There was no significant difference in ALP bioactivity of 0.07 nM insulin and control osteogenic culture.

### 3.3. Insulin Differentially Upregulates IGF-I Receptor mRNA during Osteogenic Differentiation of TPCs (Figure 3)

Osteogenic differentiation of TPCs (without insulin supplementation) did not induce upregulation of IGF-I receptor or insulin receptor mRNA. In day 14 of osteogenic cultures, both 0.07 and 0.7 nM insulin groups had significantly increased (8.1‐fold ± 1.1; *p* = 0.02; 13.3‐fold ± 1.3; *p* = 0.001) IGF-I receptor mRNA levels. In contrast, insulin supplementation to TPC osteogenic cultures did not upregulate insulin receptor mRNA level.

### 3.4. Effect of Picropodophyllin (PPP) on Insulin-Mediated Osteogenic Differentiation of TPCs (Figure 4)

#### 3.4.1. Alizarin Red Staining (Figure 4(a))

Similar osteogenic cultures of TPCs with 0.07 and 0.7 nM insulin were also maintained in the presence of 100 nM PPP, a specific inhibitor of IGF-I receptor. Addition of PPP to control osteogenic cultures did not prevent cell aggregation and secretion of mineralized matrix. However, addition of PPP to osteogenic cultures containing insulin prevented aggregation and deposition of mineralized matrix. There was minimal Alizarin Red stain uptake in day 14 of osteogenic cultures treated with PPP and 0.07 and 0.7 nM insulin.

#### 3.4.2. Osteogenic Gene Expression

Given that, 0 and 0.07 nM insulin groups were not consistently different from each other, and the effect of PPP was assessed in the 0.7 nM insulin group. In day 14 of osteogenic cultures treated with PPP and 0.7 nM insulin, mRNA levels of Runx2, ALP, and Osteocalcin were significantly reduced compared to the 0.7 nM insulin group. The mRNA levels of all 3 osteogenic markers in day 14 of osteogenic cultures treated with PPP and 0.7 nM insulin and osteogenic control group were not significantly different from each other.

#### 3.4.3. ALP Bioactivity

Consistent with transcriptional results, ALP bioactivity in day 14 of osteogenic cultures treated with PPP and 0.7 nM insulin was significantly reduced compared to insulin alone and was similar to osteogenic control.

## 4. Discussion

Given insulin/IGF-I's role in mesenchymal stem cell (MSC) osteogenesis [22, 23, 28–30], we hypothesized that insulin promotes proliferation and in vitro osteogenic differentiation of TPCs. Our results indicate that insulin significantly increased TPC proliferation after 3 days of monolayer culture. Secondly, insulin significantly increased the in vitro osteogenic capacity of TPCs compared to control osteogenic cultures, in a dose-dependent manner. These outcomes support our hypothesis. Interestingly, adding picropodophyllin, a selective IGF-I receptor inhibitor, to insulin-treated cultures mitigated the enhanced osteogenic differentiation capacity of TPCs and returned to baseline levels as control osteogenic cultures.

Our experiments were focused on evaluating the impact of sustained hyperinsulinemia or impaired insulin sensitivity on TPCs. The insulin concentrations used in this study were extrapolated from plasma insulin concentrations of people with hyperinsulinemia/insulin resistance [31, 32]. Insulin has been shown to have a pro-proliferative effect on several cell types, including mesenchymal stem/progenitor cells [33–38]. Insulin and IGF-I ligands and their respective receptors share marked similarities in their signaling properties in eukaryotic cells [22]. Insulin increased cell proliferation by binding to both insulin and/or IGF-I receptors and subsequent activation of Akt and/or ERK pathways [33, 34, 37]. Similarly, IGF-I inhibited cell cycle arrest in TPCs via the Akt pathway and consequently increased cell proliferation [39–42]. Although we have not investigated the mechanism involved in pro-proliferative effects of insulin on TPCs, similar mechanisms are likely involved. On the other hand, Lin et al. demonstrated that high glucose decreases in vitro TPC proliferation and induces apoptosis [21]. A subsequent study by the same group showed that TPCs isolated from rats with experimental diabetes had a decreased rate of in vitro proliferation compared to control TPCs. In both studies, hyperglycemia alone as a pathophysiologic stimulus in DM on characteristics of TPCs was evaluated. In light of these findings, assessing the combined effects of high insulin and high glucose on TPC viability/proliferation is warranted.

Insulin plays a critical role in skeletal bone formation and maintenance [43]. Insulin stimulates endogenous IGF-I signaling by binding to IGF-I receptor, also a key factor for osteogenic differentiation and bone development [44, 45]. In this study, insulin enhanced the TPC osteogenic capacity by upregulating mRNA expression of Runx2, a master transcriptional regulator of osteogenic differentiation, in a dose-dependent manner. Runx2 upregulation is necessary for subsequent expression of critical matrix proteins such as collagen type I, Osteocalcin, and Osteopontin. The osteogenic differentiation and mineralization mediated by IGF-I ligand demonstrated in other MSC types occurs via activation of Akt and/or ERK pathways [22, 23, 34, 46]. Given that insulin and IGF-I activate similar intracellular signaling pathways, insulin potentially enhances TPCs' osteogenic capacity via activation of Akt and/or ERK pathways.

Our data indicates that insulin supplementation (at the concentrations used in this study) during TPC osteogenesis differentially upregulates IGF-I receptor mRNA expression without marked changes in insulin receptor mRNA expression. To this end, PPP added to insulin-treated TPC osteogenic cultures prevented the increased osteogenic differentiation (osteogenic mRNA levels, mineralized matrix secretion assessed with Alizarin Red staining, and alkaline phosphatase bioactivity) of TPCs. Since PPP did not interfere with the basal osteogenic capacity of TPCs, a complex interplay between conditions favoring ossification at the expense of tenogenesis is likely. A recent study by Jiang et al. demonstrated that IGF-I potentiated the osteogenic differentiation induced by BMP9 in bone marrow-derived MSCs [47]. Accepting the role of bone morphogenetic protein (BMP) signaling dysregulation (upregulated BMP receptor-1a, BMP receptor 2, Smad1, and Smad5 mRNAs) in tendon pathological ossification and TPCs undergoing in vitro and in vivo osteogenesis [12, 48, 49], assessing BMP signaling in insulin treated TPCs is warranted.

The clinical importance of these in vitro outcomes requires further investigation, given that impaired insulin sensitivity/hyperinsulinemia is frequently present in combination with hyperglycemia in diabetic patients. With regard to TPC proliferation, our study, among others, demonstrates that hyperinsulinemia is pro-proliferative, whereas hyperglycemia decreases proliferation. In contrast, the overall osteogenic capacity of TPCs is increased under both pathophysiologic stimuli of DM (high insulin and high glucose) and may support TPC-mediated pathological ossification. Accepting the critical roles of tendon ECM and mechanical loading on tendon homeostasis and TPC bioactivity, these outcomes will need to be confirmed in vivo. Impaired insulin sensitivity alone or in combination with hyperglycemia represents an important trigger that underlies degenerative processes in cardiovascular tissues, specifically, disturbed ECM assembly leading to calcification diabetic patients. Although the mechanisms resulting in aberrant TPC phenotypes under inflammatory/healing conditions differ widely, these experiments represent the first step in determining the effect of hyperinsulinemia on TPC activity and phenotype.

## 5. Conclusion

In summary, insulin increased the proliferation and osteogenic capacity of flexor tendon-derived progenitor cells in vitro. Addition of picropodophyllin, a selective IGF-I receptor, mitigated the increased osteogenic capacity of TPCs, indicating that IGF-I signaling plays an important role in insulin-mediated osteogenic differentiation of TPCs. These findings suggest that hyperinsulinemia may alter TPC phenotype and subsequently impact the quality of repair tendon tissue. Further studies are necessary to identify the cellular and biological processes involved in diabetic tendinopathy.

## Figures and Tables

**Figure 1:**
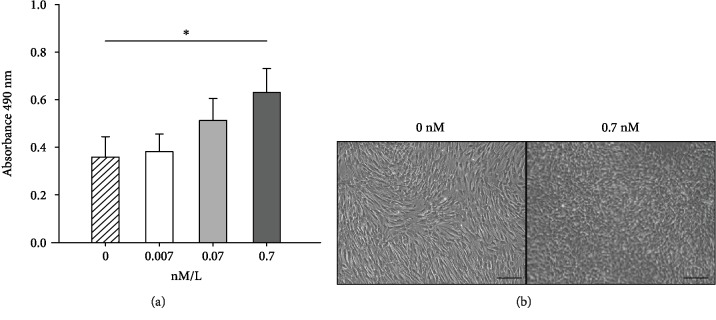
(a) TPC proliferation measured via MTT assay after 3 days of monolayer culture in basal medium supplemented with 0, 0.007, 0.07, and 0.7 nM/L insulin. ∗ represents a significant difference between 0 and 0.7 nM/L insulin (*p* ≤ 0.05). (b) Representative phase-contrast photomicrographs of TPC monolayer cultures maintained in basal medium and basal medium with 0.7 nM insulin for 3 days. A significant increase in TPCs is seen in insulin-supplemented medium. Scale bar = 100 microns.

**Figure 3:**
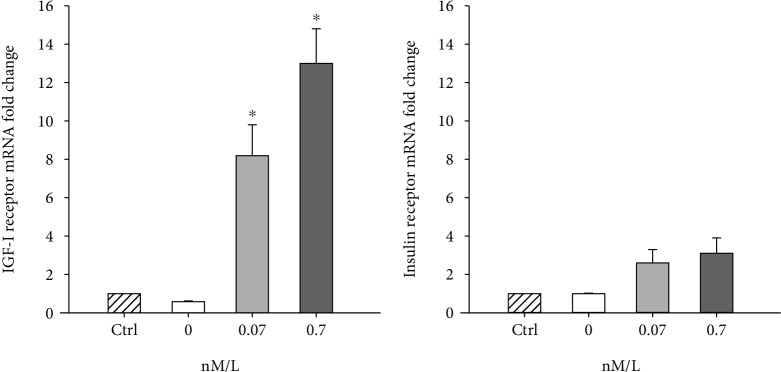
mRNA levels (normalized to EF1a) of IGF-I receptor and insulin receptor of TPC control monolayer cultures maintained in basal medium (Ctrl) and TPC osteogenic cultures supplemented with 0, 0.07, and 0.7 nM/L insulin. ∗ represents a significant increase (*p* ≤ 0.05).

**Figure 4:**
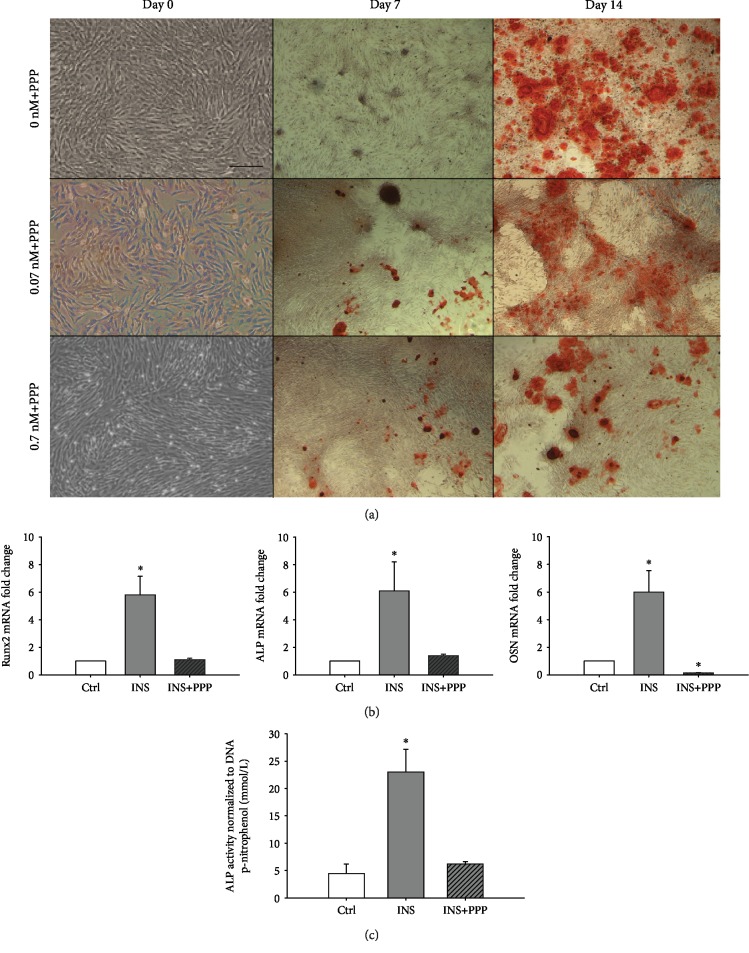
(a) Alizarin Red-stained TPC osteogenic cultures with 0, 0.07, and 0.7 nM/L insulin and 100 nM of picropodophyllin (PPP). PPP mitigated aggregation and mineralized matrix secretion of TPCs seen with insulin treatment. Scale bar = 500 microns. (b) mRNA levels (normalized to EF1a) of RUNX2, ALP, and Osteonectin (OSN) in TPC osteogenic cultures without insulin (Ctrl), supplemented with 0.7 nM/L insulin (INS) and with 0.7 nM/L insulin and 100 mM picropodophyllin (INS+PPP). PPP returned the increased osteogenic gene expression seen in insulin-treated TPCs back to baseline. ∗ represents a significant increase between treatment groups (*p* ≤ 0.05). (c) Alkaline phosphatase bioactivity (normalized to DNA) of TPC osteogenic cultures without insulin (Ctrl), supplemented with 0.7 nM/L insulin (INS) and with 0.7 nM/L insulin and 100 mM picropodophyllin (INS+PPP). ∗ represents a significant increase between treatment groups (*p* ≤ 0.05).

**Table 1:** Primers used for real-time PCR amplification.

Gene		Sequence	Amplicon (bp)
Runx2	S A	5′ CAG ACC AGC AGC ACT CCA TA 5′ CAG CGT CAA CAC CAT CAT TC	177
Alkaline phosphatase	S A	5′ TGG GGT GAA GGC TAA TGA GG 5′ GGC ATC TCG TTG TCC GAG TA	221
Osteonectin (OSN)	S A	5′ AAC CTT CTG ACC GAG AAG CA 5′ TGG GAC AGG TAC CCA TCA AT	190
Insulin receptor	S A	5′ CGA GGA CTA TCT GCA CAA TG 5′ ACC GTC ACA TTC CCG ACA TC	182
IGF-I receptor	S A	5′ TCC TAA CCC TGA CTT CGG CG 5′ TTC TTG GCA TGT CTG TGT GG	212
EF1-alpha	S A	5′ CCC GGA CAC AGA GAC TTC AT 5′ AGC ATG TTG TCA CCA TTC CA	328

## Data Availability

All data used to support the findings of this study are included within the article.
